# A Multivariate Genome-Wide Association Study Reveals Neural Correlates and Common Biological Mechanisms of Psychopathology Spectra

**DOI:** 10.21203/rs.3.rs-4228593/v1

**Published:** 2024-04-08

**Authors:** Christal Davis, Yousef Khan, Sylvanus Toikumo, Zeal Jinwala, D Boomsma, Daniel Levey, Joel Gelernter, Rachel Kember, Henry Kranzler

**Affiliations:** Crescenz VA Medical Center; University of Pennsylvania Perelman School of Medicine; University of Pennsylvania; University of Pennsylvania Perelman School of Medicine; Vrije Universiteit Amsterdam, The Netherlands; Yale School of Medicine; Yale University; University of Pennsylvania; University of Pennsylvania Perelman School of Medicine

## Abstract

There is considerable comorbidity across externalizing and internalizing behavior dimensions of psychopathology. We applied genomic structural equation modeling (gSEM) to genome-wide association study (GWAS) summary statistics to evaluate the factor structure of externalizing and internalizing psychopathology across 16 traits and disorders among European-ancestry individuals (n’s = 16,400 to 1,074,629). We conducted GWAS on factors derived from well-fitting models. Downstream analyses served to identify biological mechanisms, explore drug repurposing targets, estimate genetic overlap between the externalizing and internalizing spectra, and evaluate causal effects of psychopathology liability on physical health. Both a correlated factors model, comprising two factors of externalizing and internalizing risk, and a higher-order single-factor model of genetic effects contributing to both spectra demonstrated acceptable t. GWAS identified 409 lead single nucleotide polymorphisms (SNPs) associated with externalizing and 85 lead SNPs associated with internalizing, while the second-order GWAS identified 256 lead SNPs contributing to broad psychopathology risk. In bivariate causal mixture models, nearly all externalizing and internalizing causal variants overlapped, despite a genetic correlation of only 0.37 (SE = 0.02) between them. Externalizing genes showed cell-type specific expression in GABAergic, cortical, and hippocampal neurons, and internalizing genes were associated with reduced subcallosal cortical volume, providing insight into the neurobiological underpinnings of psychopathology. Genetic liability for externalizing, internalizing, and broad psychopathology exerted causal effects on pain, general health, cardiovascular diseases, and chronic illnesses. These findings underscore the complex genetic architecture of psychopathology, identify potential biological pathways for the externalizing and internalizing spectra, and highlight the physical health burden of psychiatric comorbidity.

## Introduction

Traditional categorical classifications of psychopathology suffer from significant limitations. In epidemiological studies, psychiatric disorders consistently co-occur more often than expected,^1,2^ suggesting overlapping genetic underpinnings.^3^ Furthermore, largely arbitrary thresholds and polythetic criterion sets yield thousands of unique symptom combinations that lead to the same diagnosis.^4^ Along with the challenges these limitations present for clinical care, they hinder progress in psychiatric genetics and neuroscience research, where efforts to identify biological mechanisms that underlie psychiatric illness have had limited success.^5,6^ Recent attempts to address these limitations have included alternative approaches to understanding psychopathology, most notably the Hierarchical Taxonomy of Psychopathology (HiTOP) and the National Institute of Mental Health’s Research Domain Criteria (RDoC) initiative.^5,7–9^ HiTOP proposes a dimensional structure of psychopathology that progresses hierarchically from symptoms to an overarching psychopathology factor. In contrast, RDoC aims to identify the underlying mechanisms of psychopathology by focusing on domains of functioning rather than diagnostic categories. Despite differences in the units of analysis and the dimensions they identify, these systems’ constructs align well in a model of psychopathology.^10^

Beginning in the 1990s, twin and family studies showed that dimensions of psychopathology had a shared genetic basis,^11,12^ with externalizing and internalizing psychopathology being the subject of much of this research. Whereas externalizing behaviors involve interaction with the social environment (e.g., aggression, impulsivity), internalizing behaviors are directed inwards (e.g., anxiety, depression).^13^ With statistical and methodological advances, molecular genetic research has also identified common externalizing^14^ and internalizing^15,16^ genetic factors that underlie each spectrum of psychopathology. Twin and family studies^17–19^ and principal component analyses^17,20^ have also examined genetic factors shared by both externalizing and internalizing psychopathology. Genome-wide association studies (GWAS) of childhood behavior problems, which encompass externalizing and internalizing psychopathology, identified two genome-wide significant loci.^21,22^

Romero and colleagues recently used a cross-trait GWAS meta-analysis to identify pleiotropic genetic effects across 12 psychiatric disorders.^23^ Because the meta-analytic signal in that study was driven by schizophrenia, the interpretation and joint biological characterization of the cross-trait signal was limited. Genomic structural equation modeling (gSEM) offers several advantages over cross-trait meta-analysis for identifying the shared genetic architecture that underlies psychopathology. First, gSEM enables specific hypotheses about the factor structure of psychopathology to be tested, with explicit comparison of proposed models that could account for the overlap observed across externalizing and internalizing spectra. Second, the use of latent variables helps to identify the common genetic effects across externalizing and internalizing spectra, minimizing the capture of genetic signals associated with the most dominant trait, as in the meta-analytic study of Romero *et al*.^23^

Other gSEM studies have investigated the factor structure of psychiatric disorders and identified one to four factors that underlie their shared liability.^16,24,25^ A previous GWAS identified two genome-wide hitsfor a higher-order *p*-factor encompassing compulsive, psychotic, internalizing, and neurodevelopmental disorders, and 66 significant hits upon *post hoc* examination of a bifactor model *p*-factor. Because the study included only psychiatric disorders, it did not capture a broad spectrum of psychopathology consistent with dimensional models like HiTOP. It also included only two internalizing (anxiety and major depressive disorder) and two externalizing (attention-deficit hyperactivity disorder and problematic alcohol use) conditions.

To conduct a detailed examination of the shared genetic architecture of externalizing and internalizing psychopathology, we applied gSEM to large GWAS summary statistics. Adopting a dimensional, transdiagnostic approach, we rst evaluated models of psychopathology to determine which factor structure provided the best fit to the pattern of genetic covariance across 16 externalizing and internalizing traits and disorders. To identify genetic effects for the externalizing spectrum, internalizing spectrum, and *across* the externalizing and internalizing spectra, we conducted GWAS on the latent factors derived from models with acceptable t. Next, we performed downstream analyses to characterize biological mechanisms underlying the shared genetic liability to psychopathology and to examine potential causal effects on physical health. Identifying mechanisms that account for vulnerability across levels of psychopathology can yield insights into the genetic basis of psychopathology, which could lead to advancements in treatment, diagnosis, and disorder classification.

## Subjects and Methods

### Summary Statistics

#### Externalizing.

Ten sets of summary statistics in European-ancestry (EUR) individuals were selected based on existing theory regarding the externalizing spectrum (Supplementary Table 1). We included summary statistics from the largest available GWAS of the following externalizing disorders: attention deficit hyperactivity disorder (ADHD; n = 225,534),^26^ four substance use disorders [SUDs; i.e., alcohol (AUD; n = 753,248),^27^ cannabis (CanUD; n = 886,025),^28^ opioid (OUD; n = 425,944),^29^ and tobacco (TUD; n = 495,005)^30^]. We also included broader measures of externalizing psychopathology [age of first sexual intercourse (AgeSex; reverse-coded; n = 317,694),^14^ general risk tolerance (Risk; n = 431,126),^14,31^ number of sexual partners (NumSex; n = 370,711),^14,31^ antisocial behavior (ASB; n = 16,400),^32^ and automobile speeding propensity (n = 404,291)^31^].

#### Internalizing.

Summary statistics from eight GWAS in EUR individuals were selected to capture the internalizing spectrum (Supplementary Table 1). Three were the largest available GWAS of internalizing disorders: (1) anorexia nervosa (AN; n = 72,517),^33^ (2) major depressive disorder (MDD; n = 1,074,629),^34^ and (3) posttraumatic stress disorder (PTSD; n = 214,408).^35^ To reflect a broad liability to internalizing, we included irritability (http://www.nealelab.is/uk-biobank/; n = 345,231), loneliness (n = 490,689),^36^ subjective wellbeing (SWB; reverse-coded; n = 298,420),^37^ miserableness (http://www.nealelab.is/uk-biobank/; n = 355,182), and anxiety (ANX; n = 280,490).^38–40 38–41^ To boost power to detect variants associated with both anxiety disorders and subclinical anxiety, we combined three anxiety GWAS^38–40^ using multi-trait analysis of GWAS.^41^

### Exploratory Analysis

#### Genetic Correlations.

Using linkage disequilibrium score regression (LDSC)^42^ in GenomicSEM,^24^ we calculated genetic correlations (*r*_g_) between the input traits. Single nucleotide polymorphisms (SNPs) were filtered using EUR HapMap3 reference panels,^43^ and SNPs with MAF < 0.01 were removed. LDSC was performed using ancestry-matched 1000 Genomes Phase 3 linkage disequilibrium (LD) scores.^44^ When available in the summary statistics, SNP-level sample sizes were specified. Otherwise, the effective sample size was calculated by summing effective sample sizes across the input GWAS cohorts.^45^ After conducting LDSC, genetic correlations were inspected to identify traits having weak associations with the other input traits prior to fitting structural equation models. Traits with average *r*_g_ < 0.20 were excluded from gSEM models.

#### Hierarchical Cluster Analysis.

To evaluate whether traits clustered with their predicted spectrum, we conducted hierarchical cluster analysis of the *r*_*g*_ matrix using the hclust() function in RStudio.^46^ We calculated a Euclidean distance matrix and used Ward’s agglomerative clustering algorithm^47^ to identify clusters. A plot of the within-cluster sum of squares was used to determine the optimal number of clusters.

#### Network Analysis.

A network analysis of the *r*_g_ matrix was performed using the *igraph* package in RStudio.^48^ The matrix was transformed into an undirected and weighted network graph, in which nodes represent each trait and the weights of the links between traits represent the magnitude of their genetic correlation. The optimal network community structure was determined by maximizing modularity, a measure of the quality of a clustering solution.

### Genomic Structural Equation Modeling

We fit four confirmatory factor analyses (CFAs) based on existing theories of psychopathology.^49–51^ First, we evaluated a correlated factors model with two factors representing externalizing and internalizing psychopathology. Next, we fit a bifactor model consisting of a general psychopathology factor on which all traits loaded, and two narrower externalizing and internalizing psychopathology factors. A higher-order model was also fit with two first-order factors representing externalizing and internalizing, and a second-order factor (EXT + INT) representing genetic effects shared by the two spectra. Finally, we fit a unidimensional, or *p*-factor, model where all traits loaded onto a single latent factor. In all CFA models, the residuals of the four SUDs were allowed to correlate; all other residuals were uncorrelated. We evaluated the models with chi-square, the Akaike information criterion (AIC), comparative fit index (CFI), and standardized root mean squared residual (SRMR) fit statistics. We also inspected the results for low (< 0.35) or negative loadings as an indicator of each model’s appropriateness.

When preparing the data for GWAS, we excluded SNPs with MAF < 0.01 and imputation scores < 0.6. Coe cients and standard error values were standardized across summary statistics to ensure that effects were scaled similarly for all traits. Each SNP was regressed on the model latent variable(s) using diagonally weighted least squares estimation. After performing GWAS, we calculated factor-specific Q_SNP_ values by comparing the fit of a common pathway model to an independent pathway model.^16^ Q_SNP_ provides a measure of SNP heterogeneity, reflecting the extent to which a SNP exerts effects entirely through the common factor (i.e., common pathway model) or, instead, exerts effects differentially across a factor’s indicators (i.e., independent pathway model). SNPs with a Q chi-square p-value < 5×10^− 8^ were filtered prior to conducting all subsequent analyses. Finally, to identify lead SNPs for each factor, we performed LD clumping in PLINK 1.9^52^ using a range of 3 Mb and *r*^2^ > 0.10 with the EUR 1000 Genomes Phase 3 reference panel.^44^

### Biological Characterization

Gene-based tests, gene-set enrichment, and gene-tissue expression analyses were conducted using MAGMA^53^ in FUMA v1.6.0^54^. We examined gene expression in BrainSpan^55^ and GTEx v8^56^ tissue samples. In FUMA, gene associations were identified based on their: (1) position, (2) expression quantitative trait loci (eQTLs) from PsychENCODE^57^ and GTEx v8^56^ brain tissue samples, and (3) chromatin interactions using Hi-C data from the dorsolateral prefrontal cortex, hippocampus, ventricles, and neural progenitor cells. We also analyzed gene expression at the cellular level in single-cell RNA sequencing (scRNA-seq) datasets from 15 human brain cell expression pro les.^58–61^ For these analyses, we used a three-step approach: (1) conducting gene-property analyses within each dataset, (2) identifying independent associations using within-dataset conditional analyses, and (3) identifying independent clusters of signals using cross-datasets conditional analyses.^58^

### Transcriptome Wide Association Studies

We conducted two transcriptome-wide association studies (TWAS) on each factor.^62^ First, we used S-MultiXcan^62^, which prioritizes likely causal genes by jointly predicting gene expression across multiple tissues. S-MultiXcan produces an overall Z-score and p-value across all tissues, as well as values for the most and least associated tissues. We examined expression across the 13 brain tissues in GTEx v8^56^ and identified the most associated tissue for each gene. To complement this approach, we used S-PrediXcan^63^ and weights trained on transcriptional differences in the frontal and temporal cortices of psychiatric cases and controls^64^ from PsychENCODE.^65^ A Bonferroni correction was applied to identify significant associations.

### Associations with Brain Phenotypes

We used BrainXcan^66^ to examine associations between the psychopathology spectra and 327 brain image-derived phenotypes (IDPs) from structural (T1-weighted) and diffusion magnetic resonance images (dMRIs) using ridge regression. Effect sizes and p-values were adjusted using LD block-based permutation, and Bonferroni correction was used to account for multiple testing (T1: 0.05/109 = 4.59 × 10^− 4^; dMRI = 0.05/218 = 2.29 × 10^− 4^). We also conducted bidirectional Mendelian randomization (MR) analyses for the most significantly associated brain IDPs. Because the signi cance of the IDP-factor association was used to identify pairs on which to perform MR, the resulting MR p-values were used to discern the possible direction of association, rather than to evaluate signi cance.^66^

### Drug Repurposing

To identify gene targets for drug repurposing, we used ve different gene annotation approaches: (1) MAGMA,^53^ (2) chromatin interactions, (3) eQTL, (4) S-MultiXcan, and (5) S-PrediXcan.^62,63^ To avoid unreliable associations, we queried the subset of druggable genes^67^ identified by multiple biological annotation sources using the Drug-Gene-Interaction Database (DGIdb).^68^ For the first-order factors, we limited drug repurposing analyses to genes that showed specificity of association with *either* externalizing or internalizing.

### Causal Mixture Models (MiXeR)

Univariate MiXeR analyses^69^ were conducted to estimate each factor’s polygenicity (i.e., the number of causal variants required to explain 90% SNP heritability) and discoverability (i.e., the average effect size of causal variants).^70^ Next, bivariate models were used to identify the proportion of unique and shared causal variants for the externalizing and internalizing spectra. In contrast to genetic correlations, MiXeR accounts for polygenic overlap regardless of whether causal variants have the same or opposite direction of effect.

### Genetic Correlations

Using the Complex Trait Genetics Virtual Lab^71^, we calculated batch genetic correlations between each factor GWAS and 1,437 phenotypes from publicly available GWAS. GWAS that were used as an input for the gSEM models were excluded. Genetic correlations were calculated using LDSC^42^ and EUR 1000 Genomes Phase 3 data^44^ as LD references. To account for multiple testing, a Benjamini-Hochberg false discovery rate (FDR) correction was applied to each set of genetic correlations.

### Generalized Summary-data-based Mendelian Randomization

To evaluate potentially causal impacts of externalizing and internalizing genetic risk on physical health, we conducted Generalized Summary-data-based Mendelian Randomization (GSMR)^72^ using 15 health traits as outcomes. Traits were chosen across four domains—(1) pain, (2) general health, (3) cardiovascular disease, and (4) chronic illness—each of which has demonstrated associations with psychopathology.^73–75^ We used summary statistics from GWAS of pain intensity,^76^ multisite chronic pain,^77^ and back pain.^78^ General health indices included GWAS summary statistics from the UK Biobank for longstanding illness, disability, or infirmity; hospitalization; and age at death. We selected ve cardiovascular GWAS: (1) heart failure,^79^ (2) stroke,^79^ (3) myocardial infarction,^80^ (4) hypertension (http://www.nealelab.is/uk-biobank/), and (5) abdominal aortic aneurysm.^79^ Finally, we selected four GWAS of chronic illnesses: (1) type 2 diabetes,^81^ (2) inflammatory bowel disease (IBD),^82^ (3) chronic obstructive pulmonary disease (COPD),^79^ and (4) asthma.^79^ Genetic instruments with significant pleiotropic effects on both the exposure and outcome were removed using the heterogeneity in dependent instruments outlier (HEIDI) method.^72^ A Bonferroni adjusted p-value was applied to identify significant effects (0.05/45 = 0.001).

## Results

**Figure 1:**
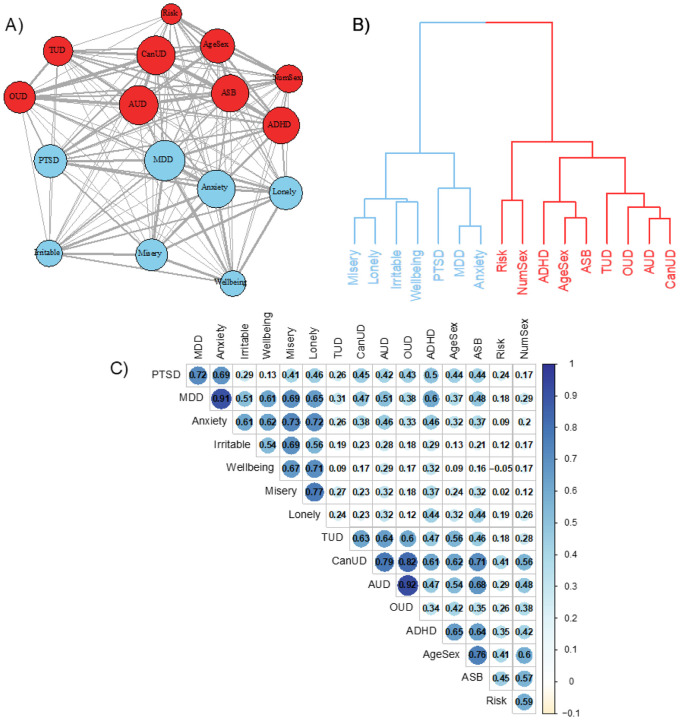
Exploratory analyses of internalizing and externalizing traits. **a)** network analysis results where colors represent clusters, the size of nodes indicates centrality, and the width of the edges between nodes represents the genetic correlation between traits, **b)** agglomerative hierarchical clustering using Ward’s criterion to identify clusters, and **c)** genetic correlations for the sixteen traits that were retained for Genomic Structural Equation Modeling. PTSD = posttraumatic stress disorder, TUD = tobacco use disorder, CanUD = cannabis use disorder, AUD = alcohol use disorder, OUD = opioid use disorderh, ADHD = attention deficit hyperactivity disorder, AgeSex = age at rst sexual intercourse (reverse coded), ASB = antisocial behavior, NumSex = number of sexual partners. Wellbeing is reverse coded.

Based on previous GWAS of externalizing and internalizing^14,15,83^ and existing theory,^7,9^ we considered a total of 18 externalizing and internalizing traits for inclusion in the analyses (Supplementary Fig. 1). We excluded two traits (automobile speeding propensity and anorexia nervosa) that were weakly associated with the others in the model (mean *r*_g_ < 0.20). Network analysis and hierarchical agglomerative cluster analysis both revealed two clusters that correspond to externalizing and internalizing spectra (see Fig. 1).

**Figure 2:**
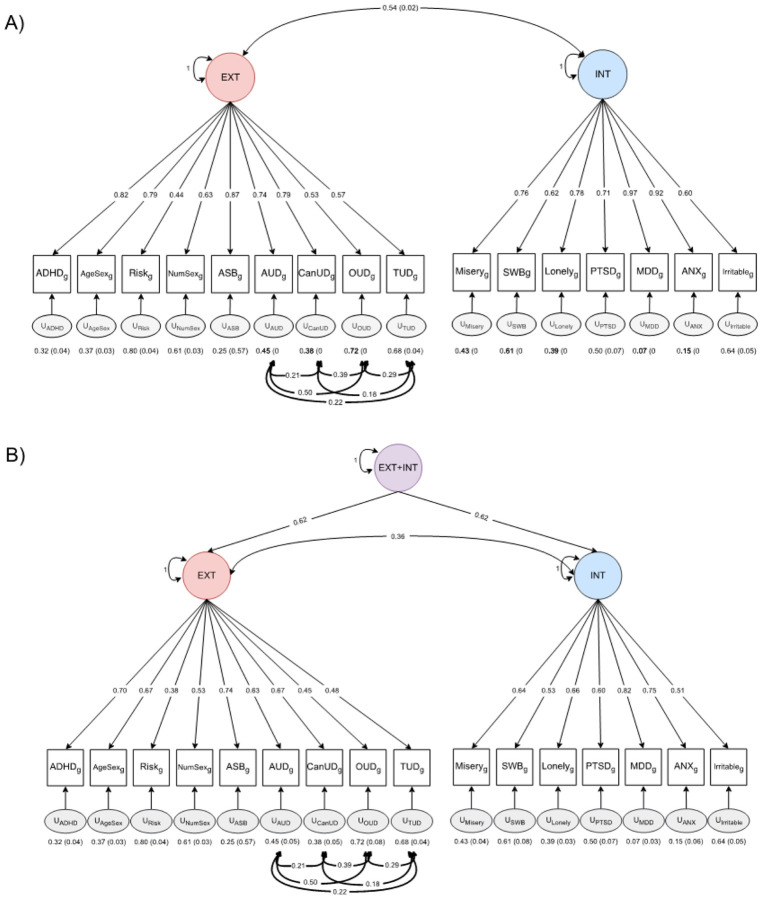
Confirmatory factor analysis models used for Genomic Structural Equation Modeling. **a)** results of the correlated factors model. Model t: X^2^(97) = 3877.82, AIC = 3955.82, CFI = 0.91, SRMR = 0.09, **b)** results of the higher order factor model. Model t: X^2^(97) = 3877.82, AIC = 3955.82, CFI = 0.91, SRMR = 0.09. EXT = externalizing, INT = internalizing, ADHD = attention deficit hyperactivity disorder, AgeSex = age at rst sexual intercourse (reverse coded), NumSex = number of sexual partners, ASB = antisocial behavior, AUD = alcohol use disorder, CanUD = cannabis use disorder, OUD = opioid use disorder, TUD = tobacco use disorder, SWB = subjective wellbeing, PTSD = posttraumatic stress disorder, MDD = major depressive disorder, ANX = anxiety. Alternative models considered can be found in Supplementary Figures 2 and 3.

Using the 16 traits, we tested several CFA models (see Fig. 2 and Supplementary Figs. 2 and 3). A general psychopathology factor model did not provide adequate fit to the data (*χ*^2^(98) = 8965.28, AIC = 9041.28, CFI = 0.79, and SRMR = 0.15), although standardized loadings were all significant and > 0.35. A bifactor model comprising a general psychopathology factor and two specific factors representing externalizing and internalizing spectra fit the data well (*χ*^2^(81) = 2527.19, AIC = 2637.19, CFI = 0.94, and SRMR = 0.05). However, the model led to several weak (< 0.35) and one negative standardized loading, possibly from overfitting the data. Thus, despite its good fit, we did not perform GWAS on factors from this model because they would be difficult to interpret. Two CFA models provided adequate fit and had strong factor loadings: (1) a correlated-factors model and (2) a higher-order factor model. Fit statistics for both models were *χ*^2^(97) = 3877.82, AIC = 3955.82, CFI = 0.91, and SRMR = 0.09. To ensure identification in the higher order model, the loadings of externalizing and internalizing onto the second-order factor were constrained equal to the square root of the genetic correlation between the externalizing and internalizing factors.^84^

### Multivariate GWAS of Psychopathology Spectra

Using a Q_SNP_ analysis, 228 independent SNPs exhibited heterogeneous effects across the externalizing spectrum (Supplementary Fig. 4). Among the associations of these SNPs within the input GWAS (Supplementary Table 2), a plurality was most strongly associated with age at rst sexual intercourse (37.23%), followed by TUD (23.38%). After filtering heterogenous SNPs, a multivariate GWAS of externalizing identified 409 GWS independent lead SNPs (Supplementary Table 3). Of these, 92 (22.49%) were not identified or within ± 1000 kb of SNPs identified by any of the input GWAS, and four were not previously associated with any externalizing trait using the same threshold. Three of the four novel SNPs were on chromosome 4 (rs1961547, rs9316, and rs7682762), with the fourth on chromosome 22 (rs1473811). These SNPs showed phenotypic associations with chronotype, schizophrenia, and social support, among other traits (Supplementary Table 4).

For internalizing, 222 independent SNPs exhibited significant heterogeneity (Supplementary Fig. 5), with most (86.49%) showing the strongest associations with MDD (Supplementary Table 5). After filtering heterogeneous SNPs, there were 85 GWS independent lead SNPs (Supplementary Table 6). Of these, 23 (27.06%) were not identified or within ± 1000 kb of SNPs identified by the input GWAS, and two were not previously associated with any internalizing trait. The two novel associations were on chromosomes 3 and 4 (rs1381763 and rs4698408, respectively). Novel SNPs had phenotypic associations with neuroticism and depression (Supplementary Table 7).

**Figure 3:**
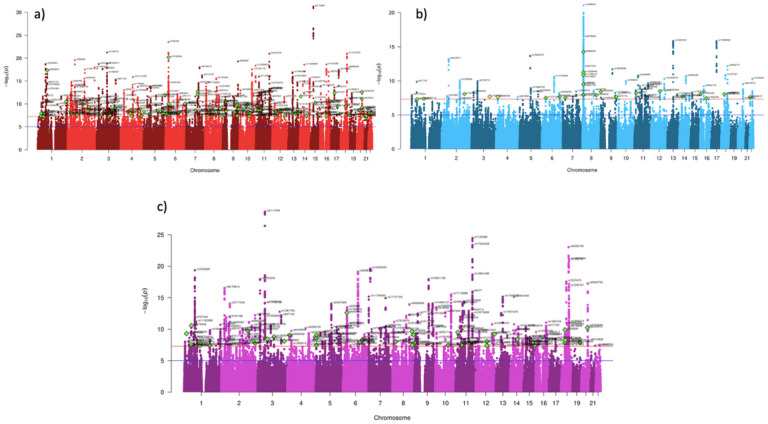
Manhattan plots of GWAS results. **a)** depicts results for the externalizing (EXT) GWAS, **b)** for the internalizing (INT) GWAS, and **c)** for the second order EXT + INT GWAS. Green diamonds denote lead SNPs not identified in any of the input GWAS for the spectrum, and yellow diamonds denote lead SNPs not previously identified in association with any of the input traits for a spectrum based on a GWAS Catalog search.

In the GWAS of genetic effects shared across externalizing and internalizing (EXT + INT factor), there were 130 lead SNPs that exhibited heterogeneous effects (Supplementary Fig. 6). Of these (Supplementary Table 8), a plurality (47.69%) was most strongly associated with age at first sexual intercourse, followed by AUD (17.69%). There were 256 GWS independent lead SNPs associated with EXT + INT (Supplementary Table 9), 38 of which (14.84%) were not identified or within ±1000 kb of SNPs identified by any of the input GWAS. All significant loci were previously associated with at least one externalizing or internalizing phenotype (Fig. 3).

### Biological Characterization

MAGMA identified 493 genes significantly associated with externalizing, including *CADM2* and *DRD2* (Supplementary Table 10). Gene-property analysis showed enriched expression during prenatal brain development (Supplementary Fig. 7). Gene expression was significantly enhanced in almost all brain tissues, with the top associations being with the cerebellar hemisphere, cerebellum, and frontal cortex (Supplementary Fig. 8). A gene-set related to mRNA binding was the only significant association with externalizing (*p*_bon_ = 0.04; Supplementary Table 11). Using scRNA-seq datasets, externalizing was significantly associated with dopaminergic and GABAergic neurons and neuroblasts from embryonic brain samples (GSE76381), human cortical neurons and hybrid cells that display characteristics of neurons and astrocytes (GSE67835), and pyramidal neurons from the cornu ammonis (CA1) hippocampal region^60^. After conditional analyses, independent significant associations remained for GABAergic neurons, cortical neurons, and hippocampal neurons (Supplementary Fig. 9).

There were 146 genes significantly associated with internalizing, including several on chromosome 8 (*BLK, XKR6*, and *C8orf12*) that were previously associated with neuroticism (Supplementary Table 12).^85^ Gene expression was not significantly enhanced at any developmental stage (Supplementary Fig. 7), but predominated in the brain, with the frontal cortex and anterior cingulate cortex most strongly associated (Supplementary Fig. 8). Although no gene-sets were significant after Bonferroni correction, the top associations were with genes involved in synaptic assembly and transmission (Supplementary Table 11). In scRNA-seq analyses, the only significant cell-type association was with GABAergic neurons (GSE76381), which was not independently significant after conditional analyses.

There were 321 genes significantly associated with EXT + INT (Supplementary Table 13). The top hits were for *FAM120AOS, DCC*, and *P4HTM*, all of which have previously been associated with both internalizing and externalizing traits. Gene expression was enhanced in brain tissue during prenatal developmental (Supplementary Fig. 7), and genes associated with the broad spectrum of psychopathology were predominantly expressed in the brain (Supplementary Fig. 8). No gene sets were significant after Bonferroni correction, but like internalizing, the top sets comprised genes involved in synaptic activity (Supplementary Table 11). Using scRNA-seq, EXT + INT showed significant associations with dopaminergic neurons (GSE76381), GABAergic neurons (GSE76381), and cortical neurons (GSE67835), though these associations were not independently significant.

### Transcriptome-Wide Association Analysis

**Figure 4:**
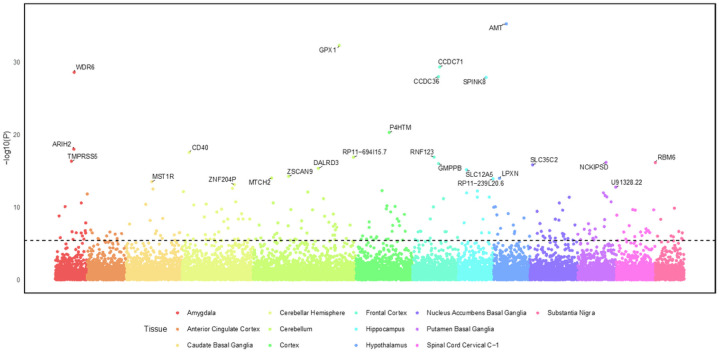
Transcriptome wide association study (TWAS) results for the second order externalizing and internalizing factor across 13 brain tissues using S-MultiXcan. The gene names for the top 25 significant associations are annotated. Signi cance was determined using a Bonferroni-adjusted p-value of 3.73 × 10–6 (0.05/13,406 tests). The dashed line at 5.43 indicates the signi cance level (−log10(3.73 × 10–6). A total of 236 associations were significant after multiple testing correction. Full TWAS results for all the factors can be found in Supplementary Tables 14–19.

Using S-MultiXcan to predict the effects of SNP variation on gene expression across 13 brain tissues revealed 352 significant gene associations for externalizing, 141 for internalizing, and 238 for EXT + INT (Fig. 4, Supplementary Tables 14–16, and Supplementary Fig. 10). TWAS using PsychENCODE data for S-PrediXcan identified 207 significant genes for externalizing, 52 for internalizing, and 124 for EXT + INT (Supplementary Tables 17–19 and Supplementary Fig. 11). Forty-five genes were identified by both S-MultiXcan and S-PrediXcan for externalizing, 21 for internalizing, and 36 for EXT + INT, with gene-property analyses showing these genes to be consistently upregulated across brain tissues (Supplementary Figs. 12–14), and three (*C1QTNF4, DPYSL5*, and SLC12A5) were almost exclusively upregulated in brain tissues.

### Associations with Brain Phenotypes

**Figure 5:**
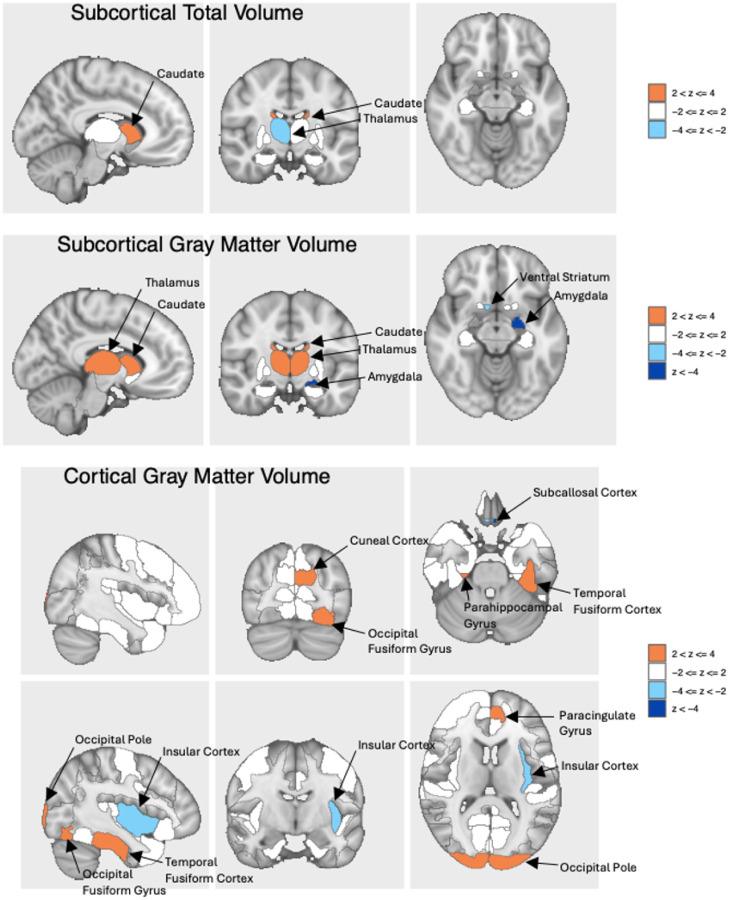
Partial results of BrainXcan association analysis for the second order externalizing + internalizing factor. Associations shown are for image-derived phenotypes from structural (T1) magnetic resonance imaging. Full results from BrainXcan are in Supplementary Figures 15–20 and Supplementary Tables 20–22. Blue colors represent reduced volume, and orange represents increased volume.

After Bonferroni correction of the LD-adjusted p-values, 8 T1 (Supplementary Fig. 15) and 12 dMRI IDPs (Supplementary Fig. 16) were significantly associated with externalizing (Supplementary Table 20), including positive associations with gray matter volume in the thalamus, caudate nuclei, and occipital pole, and negative associations with the right ventral striatum and left amygdala. From dMRIs, there were significant associations with intra-cellular volume fraction or orientation dispersion indices (ODI) in the medial lemniscus, cerebral peduncle, and middle cerebellar peduncle, among others. The only significant association for internalizing was with lower gray matter volume in the left subcallosal cortex (Supplementary Table 21 and Supplementary Figs. 17–18). Genetic factors shared across externalizing and internalizing spectra were significantly negatively associated with gray matter volume in the left amygdala and left subcallosal cortex (Fig. 5), positively associated with ODI in the medial lemniscus and left cerebellar peduncle, and negatively associated with ODI in the right external capsule (Supplementary Table 22 and Supplementary Figs. 19–20).

MR analyses showed potential bidirectional relationships between externalizing and gray matter volume in the right ventral striatum and left thalamus (Supplementary Table 23). There was greater evidence that reduced gray matter volume in the left subcallosal cortex was causally related to internalizing than vice versa (*p* = 0.008 vs. 0.401; Supplementary Table 24). Evidence was mixed regarding the direction of causal effects for the second-order externalizing and internalizing factor (Supplementary Table 25).

### Drug Repurposing

Among the 1,759 unique genes identified for externalizing using biological annotation tools, 308 were druggable targets.^67^ Sixty of these genes were identified by at least two biological annotation tools, and 52 exhibited specificity for externalizing (i.e., were not associated with internalizing). When queried in DGIdb, these genes yielded 492 drug-gene interactions (Supplementary Table 26), including with dextroamphetamine (used to treat ADHD), phenobarbital (used to prevent withdrawal symptoms from benzodiazepines and alcohol), baclofen (used to treat AUD), naltrexone (used to treat AUD and OUD), naloxone (used to reverse opioid overdose), and methadone (used to treat OUD). Gene interactions with antimigraine, anti-inflammatory, and anticonvulsant drugs (e.g., topiramate and lamotrigine) were also identified. Most of the identified drugs had regulatory approval (64.84%).

Biological annotation identified 454 unique genes associated with internalizing, 60 of which were druggable targets.^67^ Fifteen of these were identified by at least two biological annotation tools and seven exhibited specificity for internalizing, yielding 292 drug-gene interactions (Supplementary Table 27). Drug targets included antidepressants and antipsychotics. Unlike externalizing, most identified drugs (82.33%) were not currently approved, suggesting potential candidates for use in treating internalizing psychopathology.

For EXT + INT 1,138 unique genes were identified using the five biological annotation tools, nearly one-fifth (17.93%, n = 204) of which were druggable targets, with 47 of those implicated by more than one biological annotation method. Using DGIdb, we identified 460 unique drug-gene interactions (Supplementary Table 28), many of which were also present in the internalizing or externalizing results. As with internalizing, most of these drugs (75.52%) were not currently approved.

### Causal Mixture Models (MiXeR)

The externalizing and internalizing spectra displayed similar levels of polygenicity, with an estimated 12,600 and 13,200 causal variants, respectively. However, internalizing had lower discoverability $\left({{\hat{\sigma }}_{\beta }}^{2}=1.40\times 10^{-5}\right)$ than externalizing $\left({{\hat{\sigma }}_{\beta }}^{2}=1.44\times 10^{-4}\right)$, suggesting that SNPs that influence internalizing traits may exert smaller effects and thus require larger samples to detect. Despite a MiXeR-estimated genetic correlation of 0.37, almost all causal variants (96.83% of externalizing and 92.42% of internalizing; Supplementary Fig. 21) overlapped across the two spectra, with more overlap than predicted by genetic correlation alone (AIC =12.30, BIC = 4.06, where positive values indicate that the predicted model explains the data better than the genetic correlation alone). In fact, the models do not exclude the possibility that causal variants for externalizing were a subset of those for internalizing. Of the shared causal variants, 62.92% were estimated to be concordant in direction of effect.

### Genetic Correlations

Applying a Bonferroni-adjusted p-value (0.05/1368 = 3.65 × 10^− 5^), there were 413 significant genetic correlations with externalizing (Supplementary Table 29 and Supplementary Fig. 22). Tobacco phenotypes were among the most strongly correlated (current smoking: *r*_g_ = 0.79, SE = 0.02; maternal smoking around birth: *r*_g_ = 0.71, SE = 0.03; and ever smoked: *r*_g_ = 0.62, SE = 0.02), along with lower socioeconomic status, including Townsend deprivation index (*r*_g_ = 0.68, SE = 0.03), living in housing supplied by a local authority or housing association (*r*_g_ = 0.66, SE = 0.03), experiencing nancial di culties (r_g_ = 0.58, SE = 0.03), and lower educational attainment (*r*_g_ = −0.44, SE = 0.02). After Bonferroni correction, 311 phenotypes were significantly genetically correlated with internalizing (Supplementary Table 30 and Supplementary Fig. 23). Among the strongest correlations were psychiatric phenotypes, such as mood swings (*r*_g_ = 0.90, SE = 0.01), neuroticism (*r*_g_ = 0.89, SE = 0.01), and feeling fed-up (*r*_g_ = 0.82, SE = 0.01). Internalizing was also significantly genetically correlated with several types of pain (abdominal: *r*_g_ = 0.60, SE = 0.04; facial: *r*_g_ = 0.51, SE = 0.08; chest: *r*_g_ = 0.49, SE = 0.03; and multisite chronic pain: *r*_g_ = 0.49, SE = 0.03, among others). There were 474 significant genetic correlations with EXT + INT, with most being like the rst-order factors (Supplementary Table 31 and Supplementary Fig. 24).

### Generalized Summary-data-based Mendelian Randomization

**Figure 6:**
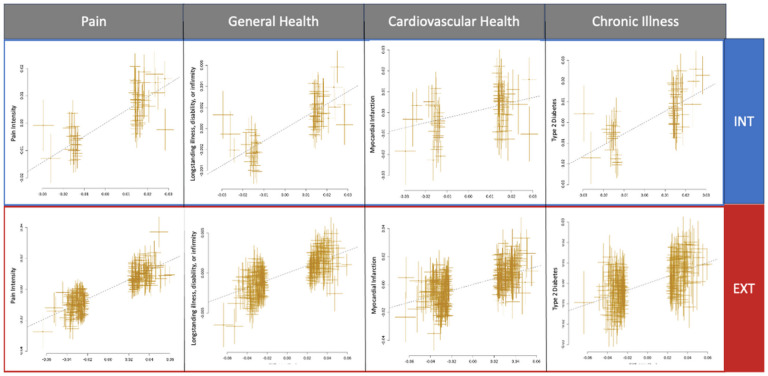
Representative results of generalized summary-data-based Mendelian Randomization (GSMR) across four domains of physical health. Pain intensity results: INT: *b*_*xy*_ = 0.48, SE = 0.04, *p* = 1.11E-28; EXT: *b*_*xy*_ = 0.31, SE = 0.01, *p* = 1.44E-155. Longstanding illness, disability, or infirmity results: INT: OR = 1.12, SE = 0.01, *p* = 9.83E-33; EXT: OR = 1.05, SE = 0.002, *p* = 2.27E-75. Myocardial infarction results: INT: OR = 1.27, SE = 0.07, *p* = 0.0003; EXT: OR = 1.24, SE = 0.02, *p* = 6.32E-38. Type 2 Diabetes results: INT: OR = 1.72, SE = 0.06, *p* = 7.28E-20; EXT: OR = 1.19, SE = 0.02, *p* = 6.94E-28. All results depicted are significant at a Bonferroni corrected p-value of 0.001. In all analyses, INT/EXT is the exposure, and the physical health trait is the outcome. INT = internalizing, EXT = externalizing, OR = odds ratio. Full results of GSMR analyses, including results for EXT+INT, can be found in Supplementary Table 32.

Externalizing had significant positive causal effects on all physical health traits examined, except age at death and IBD. Internalizing was significantly causally associated with all traits except age at death and abdominal aortic aneurysm. Additionally, internalizing had protective effects on IBD (*b*_*xy*_ = −0.32, SE = 0.09, *p* = 0.0004) and stronger positive associations than externalizing with all three pain phenotypes, all ve cardiovascular diseases, and three of four chronic illnesses (Fig. 6). Like externalizing, EXT + INT had significant positive effects on all physical health traits except age at death and IBD (Supplementary Table 32).

## Discussion

Comparing candidate factor structures of psychopathology, we found support for hierarchical models consistent with the HiTOP framework. Our models, which included symptom-level (e.g., risk tolerance and irritability) and disorder-level (e.g., TUD and MDD) manifestations of psychopathology, indicated that these traits could be organized onto higher dimensions representing externalizing and internalizing spectra, which were themselves subsumed under a broader umbrella of psychopathology genetic risk. Leveraging GWAS summary statistics that included over 1.5 million individuals, our findings also show that although there is shared variance across forms of psychopathology, the commonality does not manifest as a single overarching dimension (i.e., *p*-factor). Rather, the genetic architecture of psychopathology was better captured by a model that distinguished between specific dimensions while recognizing their interrelatedness.

Our findings also demonstrated connections between psychopathology and RDoC domains^10^ in downstream analyses that encompassed multiple RDoC units of analysis. For example, at the cellular level, externalizing-related variants were associated with RNA expression in pyramidal hippocampal neurons, which are implicated in RDoC’s cognitive control construct. For internalizing, analyses revealed molecular-level associations with drugs targeting serotonin and dopamine, which align with RDoC’s negative valence systems domain. Integrating HiTOP’s dimensional framework with RDoC’s etiological approach makes it possible to evaluate the extent to which psychopathology spectra show etiologically consistent associations across domains of functioning and units of analysis.

Although externalizing and internalizing were better represented as correlated but distinct factors and had only a small-to-moderate genetic correlation, bivariate causal mixture models estimated extensive overlap in causal variants between the two spectra. This pleiotropy suggests that there are shared biological pathways that underpin externalizing and internalizing psychopathology. Through GWAS and downstream analyses, we identified potential neural mechanisms of this shared risk. Broad psychopathology genetic risk was associated with structural and functional differences in the brain, including reduced gray matter volume in the left amygdala and subcallosal cortex. Reduced left amygdala volume has been shown to mediate the relation between childhood threat exposure and the development of externalizing and internalizing symptoms.^86^ Similarly, reduced left subcallosal cortical volume is a potential mediator of associations between personality and various emotional states (e.g., subjective well-being) and psychiatric disorders (e.g., alcohol dependence).^87^ Psychopathology liability was also related to alterations in the organization and microstructure of white matter bers, reflected by changes in orientation dispersion indices, which may signify disruptions in neural communication that contribute to various manifestations of psychopathology.^88^ In summary, we identified neural mechanisms that operate at varying levels of specificity,^89^ with some correlates linked specifically to externalizing or internalizing and others linked to broad psychopathology liability.

Extending these findings beyond mental health, MR analyses showed that liability to psychopathology exerted potentially causal effects on adverse physical health outcomes. Of note, internalizing generally showed stronger associations with pain, general health, cardiovascular disease, and chronic illness than externalizing. Previous research also supports likely causal effects of genetic risk for internalizing traits/disorders on localized pain^90,91^ and various disease outcomes.^92^ An unexpected nding here was that internalizing was protective for IBD after removing pleiotropic variants. Although some studies demonstrated causal effects of MDD on risk for IBD,^93^ studies of other internalizing disorders (e.g., anxiety) have not.^94^ Thus, more research is needed to disentangle the complex relations of internalizing and inflammatory, autoimmune conditions such as IBD. Finally, although the impact of internalizing liability on physical health was particularly pronounced, both externalizing and broad psychopathology liability also showed potentially causal effects across physical health domains. These findings underscore the contribution of psychopathology liability to the emergence of co-occurring physical health conditions.

A limitation of the present study is its inclusion of only European-ancestry individuals. Data adequate to explore a broad liability to internalizing and externalizing disorders and subclinical features in other ancestry groups were not available. Although data from GWAS of externalizing and internalizing-related disorders are available for some other non-European ancestry groups, more precise, *non-disorder* psychiatric phenotypes are limited in these populations. Nonetheless, some research suggests that a similar factor structure applies in African-ancestry individuals. At the disorder level, a gSEM of African-ancestry individuals (unpublished data) identified substance use and psychiatric disorder factors that roughly aligned with externalizing and internalizing, respectively. Additionally, the analyses showed that a higher-order factor accounted for genetic variance shared by substance use and psychiatric disorders, which to a degree corresponds to our EXT + INT factor. With the growth of diverse biobanks^95^ and deep phenotyping^96^, more direct replication in other ancestries should soon be possible and given high priority.

In conclusion, our findings supported a hierarchical structure of psychopathology, recognizing correlated externalizing and internalizing dimensions subsumed under a broader psychopathology liability. By discerning genetic variants and neural mechanisms operating at varying levels of specificity, the findings revealed the utility of applying a dimensional, hierarchical genetic approach to investigate psychopathology, which could augment existing categorical frameworks. Developing a more nuanced understanding of underlying biological mechanisms across forms of psychopathology could aid in constructing a more precise and comprehensive psychiatric nosology, providing a foundation on which to improve treatment and clinical outcomes.
